# Loop-mediated isothermal amplification as an emerging technology for detection of *Yersinia ruckeri *the causative agent of enteric red mouth disease in fish

**DOI:** 10.1186/1746-6148-4-31

**Published:** 2008-08-12

**Authors:** Mona Saleh, Hatem Soliman, Mansour El-Matbouli

**Affiliations:** 1 Clinic for Fish and Reptiles, Faculty of Veterinary Medicine, University of Munich, Germany, Kaulbachstr.37, 80539 Munich, Germany; 2 Veterinary Serum and Vaccine Research Institute, El-Sekka El-Beda St., P.O. Box 131, Abbasia, Cairo, Egypt

## Abstract

**Background:**

Enteric Redmouth (ERM) disease also known as Yersiniosis is a contagious disease affecting salmonids, mainly rainbow trout. The causative agent is the gram-negative bacterium *Yersinia ruckeri*. The disease can be diagnosed by isolation and identification of the causative agent, or detection of the *Pathogen *using fluorescent antibody tests, ELISA and PCR assays. These diagnostic methods are laborious, time consuming and need well trained personnel.

**Results:**

A loop-mediated isothermal amplification (LAMP) assay was developed and evaluated for detection of *Y. ruckeri *the etiological agent of enteric red mouth (ERM) disease in salmonids. The assay was optimised to amplify the *yruI/yruR *gene, which encodes *Y. ruckeri *quorum sensing system, in the presence of a specific primer set and *Bst *DNA polymerase at an isothermal temperature of 63°C for one hour. Amplification products were detected by visual inspection, agarose gel electrophoresis and by real-time monitoring of turbidity resulted by formation of LAMP amplicons. Digestion with *Hph*I restriction enzyme demonstrated that the amplified product was unique. The specificity of the assay was verified by the absence of amplification products when tested against related bacteria. The assay had 10-fold higher sensitivity compared with conventional PCR and successfully detected *Y. ruckeri *not only in pure bacterial culture but also in tissue homogenates of infected fish.

**Conclusion:**

The ERM-LAMP assay represents a practical alternative to the microbiological approach for rapid, sensitive and specific detection of *Y. ruckeri *in fish farms. The assay is carried out in one hour and needs only a heating block or water bath as laboratory furniture. The advantages of the ERM-LAMP assay make it a promising tool for molecular detection of enteric red mouth disease in fish farms.

## Background

Yersiniosis or enteric red mouth disease (ERM) is a serious systemic bacterial infection of fishes which causes significant economic losses in salmonid aquaculture worldwide [1]. Although infection with this agent has been reported in other fish species, salmonids especially rainbow trout *Oncorhrynchus mykiss*, are highly susceptible to ERM [2,3]. The disease was first described in the rainbow trout in the United State in 1958, from Hagerman Valley, Idaho by Rucker [4], and later the causative organism named *Yersinia ruckeri *[5]. The disease is endemic in North America [3] and widespread elsewhere. It was also described in 1981 in France, Germany and United Kingdom and has now been reported in most of Europe, Australia [6,7] and South Africa [8].

The causative agent, *Yersinia ruckeri*, is a gram-negative, non-spore-forming rod-shaped bacterium with rounded ends and like the other members of the Enterobacteriaceae family is glucose-fermentative, oxidase-negative and nitrate-reductive [9,10]. ERM outbreaks usually begin with low mortality, and then escalate to result in high losses. Characteristic symptoms of ERM are haemorrhages of the mouth and gills, though these are rarely seen in acute infections but may be present in chronic infections, diffuse haemorrhages within the swim bladder, petechial haemorrhage of the pyloric caecae, bilateral exophthalmia, abdominal distension as a result of fluid accumulation, general septicaemia with inflammation of the gut, the spleen is often enlarged and can be almost black in colour [4]. Transmission occurs by direct contact with carrier fish, other aquatic invertebrates and birds [4,11]. The ability of *Y. ruckeri *to survive and remain infective in the aquatic environment is considered to be a major factor in spread of the disease. Furthermore, *Y. ruckeri *is able to form biofilms and grow on surfaces and solid supports in fish tanks, like many bacteria in aquatic environments, which lead to recurrent infections in rainbow trout farms [12]. Although vaccination has for a decade been very successful in the control of infections caused by *Y. ruckeri *in trout farms [13], cases of yersiniosis have been reported in trout farms where vaccination didn't provide enough protection against the infection [14] and due to carrier state [13]. Different diagnostic methods have been developed for detection of *Y. ruckeri *including culturing, serological and molecular techniques. Isolation and identification using agar media and the organism's biochemical characteristics are considered the gold standard for *Y. ruckeri *diagnosis. Serological methods for detection of *Y. ruckeri *have also been developed and these include ELISA, agglutination, and the immunofluorescence antibody technique (IFAT) [15]. Molecular techniques are able to detect low levels of the bacterium and facilitate detection of asymptomatic carriers, which is very important for prevention of ERM transmission and spread [16]. Restriction fragmentation-length polymorphism [17] and PCR assays [18-20] are widely used for detection of low levels of *Y. ruckeri *in infected trout tissues and blood and also for detection of asymptomatic carriers. Although PCR has been shown to be a powerful and sensitive tool in detection of *Y. ruckeri*, its requirements for expensive equipments, a precision thermocycler and laboratory training limit its use in the field as a routine diagnostic tool.

Alternate isothermal nucleic acid amplification methods, which require only a simple heating device, have been developed to offer feasible platforms for rapid and sensitive detection of a target nucleic acid. These include nucleic acid-based amplification (NASBA), loop-mediated isothermal amplification (LAMP) and ramification amplification [21-23]. LAMP is a nucleic acid amplification method that synthesises large amounts of DNA in a short period of time with high specificity [22,24]. The strand displacement activity of *Bst *DNA polymerase impels auto-cyclic DNA synthesis with loop-forming primers to yield long-stem loop products under isothermal conditions: 60–65°C for about 60 min [22,25]. The LAMP reaction requires four or six primers that target six or eight separate DNA sequences on the target and give the assay very high specificity [22,25]. LAMP amplification products can be detected by gel electrophoresis, by real time monitoring of turbidity with a turbidimeter [24,26] or with the naked-eye. Visual detection can be accomplished using different methods such as detection of a white precipitate (magnesium pyrophosphate), use of an intercalating DNA dye such as SYBR Green I gel stain [27], use of florescent detection reagent, FDR, [28], or use of oligonucleotide probes labelled with different fluorescent dyes and low molecular weight cationic polymers such as polyethylenimine, PEI [29].

LAMP-based assays have been developed for numerous aquaculture animal pathogens, including white spot syndrome virus [30], yellow head virus [31], *Edwardsiella tarda *[32] and *Nocardia seriolae *[33], *Tetracapsuloides bryosalmonae*, *Myxobolus cerebralis*, *Thelohania contejeani *[34-36], Koi herpes virus (CyHV-3) and viral hemorrhagic septicaemia (VHS) [27,37]. The objective of this study was to develop and evaluate LAMP, as a simple, rapid and sensitive diagnostic tool for ERM disease.

## Methods

### Bacteria

The bacterial strains used in this study were listed in (table 1). *Y. ruckeri *strains were cultured on trypticase-soy-agar [3]. The purity of the cultures was tested with Gram stain and confirmed biochemically with the API 20E rapid identification system.

**Table 1:** Bacterial species assayed in ERM-LAMP experiments

**Bacterial Strains**	**Source**
*Y. ruckeri*	DSMZ^1 ^18506 (ATCC 29473)
*Y. ruckeri*	CECT^2 ^955
*Y. ruckeri*	CECT 956
*Y. ruckeri*	Dr. Joachim Nils^3^
*Y. aldovae*	DSMZ 18303 (ATCC 35236)
*Y. enterocolitica*	DSMZ 4780 (ATCC 9610)
*Y. frederiksenii*	DSMZ 18490 (ATCC 33641)
*Y. intermedia*	DSMZ 18517 (ATCC 29909)
*Y. kristensenii*	DSMZ 18543(ATCC 33638)
*Aeromonas salmonicida*	Clinic for Fish and Reptiles
*Aeromonas sorbia*	Clinic for Fish and Reptiles
*Renibacterium salmoninarum*	Clinic for Fish and Reptiles
*Flavobacterium columnare*	Clinic for Fish and Reptiles
*Pseudomonas aeroginosa*	Clinic for Fish and Reptiles

^1) ^DSMZ: Deutsche Sammlung von Mikroorganismen und Zellkulturen GmbH (German Collection of Micro-organisms and Cell Cultures) Braunschweig, Germany.

^2) ^CECT: Colección Española de Cultivos Tipo (Spanish Type Culture Collection) Valencia, Spain.

^3) ^Fischgesundheitsdienst im Staatlichen Untersuchungsamt, Veterinäruntersuchungsamt Mittelhessen, Giessen, Germany.

Each strain from other bacterial strains was propagated on its specific medium and then tested by Gram stain and biochemically.

### DNA extraction

DNA was extracted from bacterial cultures using QIAamp^® ^DNA mini kit (QIAGEN, Hilden, Germany). Bacterial cells were harvested in a microcentrifuge tube by centrifugation at 5000 × *g *for 10 min. Cell pellets were re-suspended in 180 μl lysis buffer (20 mg/ml lysozym; 20 mM Tris-HCl, pH 8.0; 2 mM EDETA; 1.2% Triton) and incubated at 37°C for 30 min. Proteinase K and Buffer AL were then added and mixed by vortexing. After 30 min incubation at 56°C, ethanol was added and thoroughly mixed to yield a homogenous solution. DNA was then extracted as per manufacturer's instructions. DNA was extracted from tissue samples (liver, kidney, spleen) by QIAamp^® ^DNA mini kit (QIAGEN, Hilden, Germany) according to the manufacturer's instructions following the animal tissues protocol.

### Oligonucleotide primers

ERM-LAMP primers were designed according to the published sequence of *yruI/yruR *(GenBank accession number AF274748, [20]) using Primer Explorer version 4 (Net Laboratory, Tokyo, Japan). Five primers were constructed; two outer primers F3 and B3, two inner primers: forward inner primer (FIP) backward inner primer (BIP) and loop forward primer (LF). FIP comprised the F1c sequence complementary to F1, a TTTT linker, and F2 sequence. BIP consisted of the B1c sequence complementary to B1, a TTTT Linker and B2 sequence. After modification of the 3' end with Rox, the loop forward primer LF was used as an Oligo DNA Probe (ODP). PCR specific primers IF-2 and IR-2 were used to amplify 1000 bp of *yruI/yruR *genes of *Y. ruckeri *[20]. Details of the LAMP and PCR primers are given in (Table 2).

**Table 2:** Details of oligonucleotide primers used for ERM-LAMP assay and PCR assay.

**Primer name**	**Length**	**Sequence (5'-3')**
F3	20-mer	TCGATATAGTTACCTTCCGG
B3	18-mer	ATGGGCAGTGAACTGTAG
FIP	46-mer	TGTTCGTTTATTGAACTTCACCGATTTTCGTCGAACTGAGCGTTAA
BIP	50-mer	AAGCTGATTTCCATAAATTCCGAGTTTTTAATGACATGGAGTTTGATGAG
Loop Forward(LF)	25-mer	AGGTATCGTGTGTTAGGATTATCGT
ODP	25-mer	AGGTATCGTGTGTTAGGATTATCGT-Rox
IF-2	24-mer	GAGCGCTACGACAGTCCCAGATAT
IR-2	24-mer	CATACCTTTAACGCTCAGTTCGAC

### Optimization of ERM- LAMP condition

ERM-LAMP reactions were carried out in a Loopamp real-time turbidimeter (LA-200, Teramecs Co., Ltd., Kyoto, Japan) at 60, 63 and 65°C, for 30, 45 and 60 min, followed by 80°C for 2 min to terminate the reaction. The reaction mixture contained 40 pmol each of inner primers FIP and BIP, 5 pmol each of outer primers F3 and B3, 20 pmol of LF (forward loop primer), 1.4 mM of dNTP mix, 1.6 M betaine (Sigma-Aldrich, GmbH, Schnelldorf, Germany), 4.5 mM MgSO_4_, 8 U of *Bst *DNA polymerase (New England Biolabs GmbH, Frankfurt, Germany), 1× of the supplied Thermopol buffer, and a specified amount of template DNA in a final volume of 25 μl. Reaction mix without DNA template was included as a negative control.

### PCR amplification

Amplification was performed in a 50 μl reaction volume with 2× ready mix PCR Master mix (Thermo Scientific, Hamburg, Germany) which contained (75 mM Tris-HCl (pH 8.8), 20 mM (NH_4_)_2 _SO_4_, 1.5 mM MgCl_2_, 0.01% Tween-20, 0.2 mM each nucleotide triphosphate, 1.25 U thermoprime plus DNA polymerase, and red dye for electrophoresis), 1.5 μl of DNA template and 20 pmol each of forward and reverse primers. The amplification was carried out in Mastercycler Gradient thermocycler, Eppendorf, with the following cycling profile: 94°C for 2 min, then 40 PCR cycles of 92°C for 1 min (DNA denaturation), 65°C for 1 min (primer annealing) and 72°C for 1.5 min (DNA extension), with a terminal extension step of 72°C for 5 min.

### Detection of the amplification products

Three detection methods were used: real-time turbidity detection, agarose gel analysis and visual detection. Changes in absorbance at 650 nm were measured for real-time turbidity detection with a Loopamp real-time turbidimeter (LA-200). A cut off value was determined based on the mean of the negative detection control optical density. Specimens with an optical density of less than 0.1 were determined to be negative for *Y. ruckeri *bacterial DNA. LAMP and PCR amplification products were analysed by gel electrophoresis stained with GelRed™ Nucleic Acid Gel Stain, 10,000× in water (BIOTREND Chemikalien GmbH, Köln, Germany) and then visualised under UV light. A TrackIt™ 100 bp DNA ladder (Invitrogen GmbH, Karlsruhe, Germany) was used as molecular weight marker. Visual detection of the LAMP products was carried out either by using 1 μl of Fluorescent Detection Reagent, FDR, (Eiken Chemical Co., Ltd) added before incubation of the reaction mixture at 63°C, or by addition of 1 μl of 1:10 diluted SYBR Green I nucleic acid gel stain 10,000 × concentration in DMSO (Cambrex BioSceince, Rockland, Inc., ME, USA) to the LAMP product after termination of the reaction. Any colour changes of the reaction mixture were noted. For detection with Rox- labelled probe, 0.2 μmol of low molecular weight PEI (Wako chemical GmbH, Neuss, Germany) was added to the LAMP product after centrifugation for 10 s at 6000 rpm to form insoluble PEI-amplicon complex, containing the Rox- labelled probe, which was precipitated by additional centrifugation at 6000 rpm for 10 s. Reaction tubes were then visualised under a conventional UV illuminator or by fluorescence microscopy.

### Restriction analysis digestion of the ERM- LAMP products

To confirm the structure of the LAMP amplicons, it was purified using a High pure PCR purification kit (Roche Molecular Biochemicals, Mannheim, Germany) and then subjected to digestion with restriction enzyme *Hph*I (New England BioLabs GmbH, Frankfurt, Germany). Fragment sizes were analyzed by 2% agarose gels electrophoresis stained with GelRed™ Nucleic Acid Gel Stain, 10,000× in water (BIOTREND Chemikalien GmbH, Köln, Germany) and then visualised under UV light.

### ERM- LAMP assay specificity

DNAs from *Y. ruckeri strains *and from other bacterial strains (*Y. aldovae, Y. enterocolitica, Y. frederiksenii, Y. intermedia, Y. kristensenii, Aeromonas salmonicida, Aeromonas sorbia, Pseudomonas aeruginosa, Renibacterium salmoninarum and Flavobacterium columnare*) were tested by ERM-LAMP assay to assess the specificity of the constructed primers. DNA from non-infected fish tissues and a negative LAMP reaction control were used to detect any non-specific amplification.

### Sensitivity of the ERM-LAMP assay

One microgram genomic *Y. ruckeri *DNA was 10-fold serially diluted to assess the lower detection limit of the LAMP assay compared with conventional PCR. The products were analysed visually and by 2% agarose gel electrophoresis.

### Feasibility of the ERM- LAMP assay

The use of the ERM-LAMP assay to detect *Y. ruckeri *DNA in clinical specimens was evaluated by testing 15 rainbow trout samples infected with ERM submitted to our clinic and 4 control fish samples. These fish were suffering from diffuse haemorrhages in the swim bladder and enlarged black spleen. The samples were tested by both ERM-LAMP assay and PCR assay.

## Results

**Figure 1:**
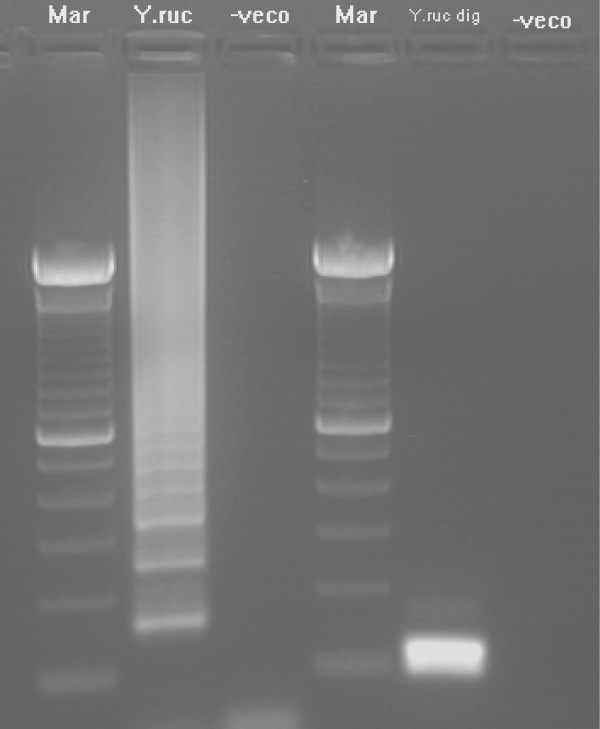
**ERM-LAMP**. *Yersinia ruckeri *loop-mediated isothermal amplification (ERM-LAMP) products and restriction analysis of ERM- LAMP product with *Hph*I enzyme. Lane Mar = 100-base-pair DNA ladder, lane Y. ruc = Amplified *Y. ruckeri *LAMP product shows a ladder-like pattern, lane Y. ruc dig = Digested *Y. ruckeri *LAMP product with *Hph*I with production of 87 bp and 108 bp bands, lane – veco = Negative (No template) control.

**Figure 2:**
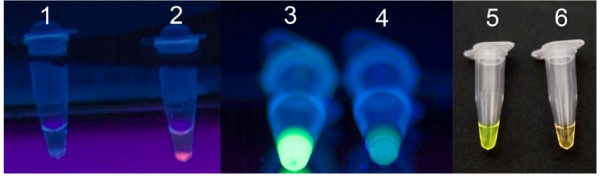
**Visual detection of ERM-LAMP product**. Using different naked eye detection methods: 1 = Negative control of ERM-LAMP reaction using Rox- labelled probe, there is neither pellet nor red fluorescence; 2 = Positive ERM-LAMP reaction using Rox- labelled probe, the pellet emitted red fluorescence; 3 = positive sample by using FDR, emitted strong green fluorescence when exposed to UV light; 4 = negative sample by using FDR, did not emitted strong green fluorescence under UV light; 5 = positive sample with green colour by using SYBR green I stain; 6 = negative sample with orange colour by using SYBR green I stain.

Optimal amplification of the *Y. ruckeri yruI/yruR *gene by ERM-LAMP assay was obtained at 63°C for 60 min, as shown by both agarose gel electrophoresis and real time turbidity measurements. Amplified products exhibited a ladder-like pattern on the gel (Fig. 1). Specificity of the amplification was confirmed by digestion of the LAMP products using *Hph*I restriction enzyme (Fig. 1), the sizes of the resultant digestion products were as predicted (87 bp and 108 bp). Results obtained with the visual detection methods correlated with agarose gel electrophoresis results. When FDR used, a strong green fluorescence was emitted by LAMP positive reactions (F ig. 2, Tube No.3) when exposed to UV light and no fluorescence was evident for a negative reaction (Fig. 2, Tube No. 4). Likewise, after addition of SYBR Green I dye, the ERM-LAMP products appeared green (Fig. 2, Tube No. 5), while in the negative control tube the original orange colour of SYBR Green I did not change (Fig. 2, Tube No. 6). With Rox-labelled probe, a pellet formed emitted a red fluorescence for a positive reaction (Fig. 2, Tube No. 2), but there was neither pellet nor fluorescence observed in the negative control tube (Fig. 2, Tube No. 1).

**Figure 3:**
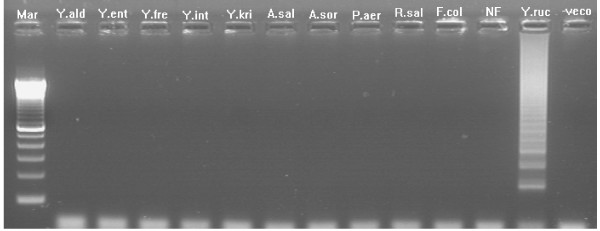
**Specificity of ERM-LAMP primers for detection of *Y. ruckeri *DNA**. Lane Mar = 100-base-pair DNA ladder, lane Y. ald = DNA from *Yersinia aldovae*, lane Y. ent = DNA from *Yersinia enterocolitica*, lane Y. fre = DNA from *Yersinia frederiksenii*, lane Y. int = DNA from *Yersinia intermedia*, lane Y. kri = DNA from *Yersinia kristensenii*, lane A. sal = DNA from *Aeromonas salmonicida*, lane A. sor = DNA from *Aeromonas sorbia*, lane P. aer = DNA from *Pseudomonas aeruginosa*, lane R. sal = DNA from *Renibacterium salmoninarum*, lane F. col = DNA from *Flavobacterium columnare*, lane NF = DNA from non-infected Fish tissues, lane Y. ruc = DNA from *Yersinia ruckeri*, lane – veco = Negative control.

**Figure 4:**
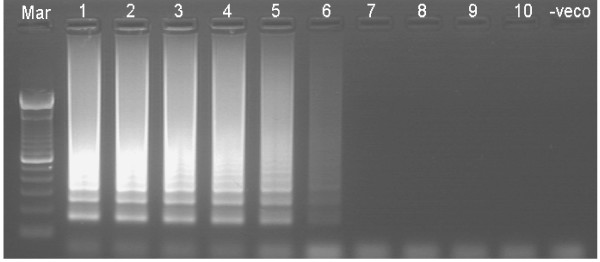
**Sensitivity of ERM-LAMP assay**. Lower detection limit of the *Yersinia ruckeri *DNA by LAMP assay. Lane Mar = 100-base-pair DNA ladder, lane 1–10 = 10-fold serial dilution of 1 μg *Yersinia ruckeri *DNA from 10^-1^-10^-10^; lane – veco = No template control.

**Figure 5:**
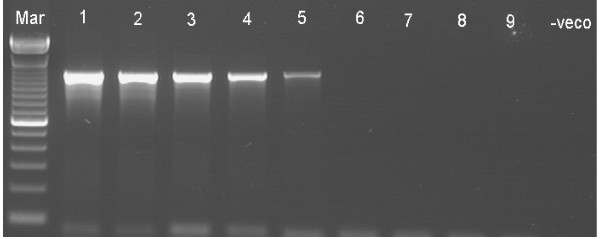
**Sensitivity of ERM-PCR assay**. Lower detection limit of (1000 bp fragment) *Yersinia ruckeri *DNA by PCR. Lane Mar = 100-base-pair DNA ladder, lane 1–9 = 10-fold serial dilution of 1 μg *Yersinia ruckeri *DNA from 10^-1^-10^-9^; lane – veco = No template control.

**Figure 6:**
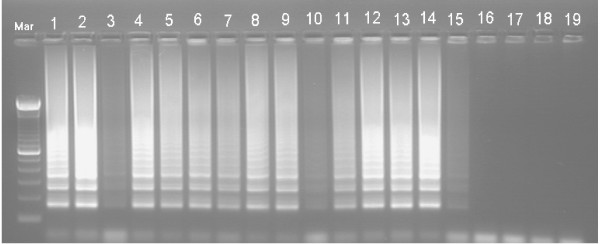
**Feasibility of ERM-LAMP assay**. Detection of *Yersinia ruckeri *DNA from 15 infected kidney samples by ERM-LAMP while there is no amplifications appeared with the non-infected kidney samples. Lane Mar = 100-base-pair DNA ladder, lanes 1–15 = DNA from infected kidney samples, lanes 16–19 = DNA from non-infected kidney samples.

**Figure 7:**
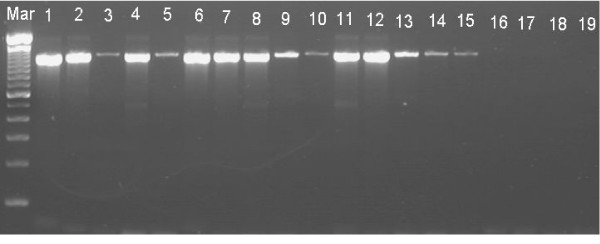
**Feasibility of ERM-PCR assay**. Detection of *Yersinia ruckeri *DNA from 15 infected kidney samples by ERM-PCR while there is no amplifications appeared with the non – infected kidney samples. Lane Mar = 100-base-pair DNA ladder, lanes 1–15 = DNA from infected kidney samples, lanes 16–19 = DNA from non-infected kidney samples.

The specificity of ERM-LAMP primers was confirmed by amplification of *yruI/yruR *gene from all *Y. ruckeri *tested strains while there are no amplification products detected from the other bacterial species, non-infected fish tissues or negative (no template) LAMP reaction control (Fig. 3). Both agarose gel electrophoresis and visual detection methods showed that, the lower detection limit of the ERM- LAMP method is 10^-6 ^dilution, which equal to 1 pg of the *Y. ruckeri *genomic DNA (Fig. 4), while PCR showed no amplification after a dilution of 10^-5 ^which equal to 10 pg *Y. ruckeri *genomic DNA (Fig. 5). The LAMP assay detected *Y. ruckeri *DNA from 15 infected fish samples, which were also positive by PCR (Fig. 6 &7). Samples from all 4 control fish were negative in both assays.

## Discussion

Efficient, rapid and timely diagnosis is critical for successful management of diseases in aquaculture. For field diagnosis, the optimal detection system should be economical, quick, and easy to operate, moreover should meet the requirements of specificity and sensitivity [38]. ERM disease is a serious infection that causes sever economic losses in salmonid aquaculture. It usually occurs as an acute condition with high morbidity and mortality rates, which necessitates rapid and accurate methods for detection of its causative agent, *Y. ruckeri *[18]. A traditional microbiological approach for isolation and identification usually takes 2 to 3 days, and given that different numerical profiles for *Y. ruckeri *can be obtained with commercial multi-substrate identification systems, particularly the API 20E system, they must be interpreted with caution [3]. Although PCR assays are more accurate, specific, and faster than the microbiological approach [18-20], they require precision equipments which are beyond the capacity of most diagnostic sites to purchase, maintain and operate, and the complexity of the assay procedures obviates the possibility of point-of-care use.

In this study, a rapid and sensitive diagnostic system based on LAMP technology was developed to detect *Y. ruckeri*. The ERM-LAMP assay requires only a simple water bath or heating block to incubate the reaction mixture at 63°C for 1 hr before the reaction products are visualised. The assay utilizes a single DNA polymerase that is active at relatively high isothermal amplification temperatures, which diminishes the probability of non-specific priming [39]. The *yruI/yruR *quorum sensing system encoding gene of *Y. ruckeri *was chosen as a suitable target, as it controls virulence gene expression through cell to cell communication and has great potential for rapid and specific identification of this fish pathogen [20]. Although there is a serotypic diversity among *Y. ruckeri *strains [40,41], *yruI/yruR *gene was amplified from all *Y. ruckeri *tested strains by PCR and produced one RFLP pattern which demonstrate a high degree of genotypic homogeneity among *Y. ruckeri *strains regarding this gene [20].

A LAMP assay requires at least 4 highly specific primers to distinguish six distinct regions on the target DNA [42]. In developing the ERM-LAMP assay, several primer sets were appraised, with the most effective set presented here. The assay was optimized to amplify *Y. ruckeri *at 63°C using a set of 4 or 5 primers. In initial trials of the assay, a characteristic ladder-like pattern of LAMP amplification is demonstrated upon gel electrophoresis [43] and confirmed the identity of the product by *Hph*I digestion. The ERM-LAMP assay was able to amplify the target *yruI/yruR *gene from all *Y. ruckeri *tested strains while it did not show any cross-reactivity with a panel of DNAs from other Yersinia species or from other related bacterial species, which confirm its specificity. Due to the isothermal nature of the LAMP assay, there is no time lost in temperature cycling, which leads to extremely high efficiency compared with regular PCR [22,44]. Another advantage of LAMP is that real-time monitoring of the reaction is possible [24] and this decreases the time needed to get results and reduces the risk of carry-over contamination in the post-PCR process [45]. Alternatively, LAMP reaction products can be visualized using SYBR Green I nucleic gel stain which has high binding affinity to double stranded DNA and hence turns from orange to green as the LAMP amplicons are produced [46,47]. LAMP product can also be monitored by placing a reaction tube directly on a UV transilluminator; when the FDR added into the reaction mixture. The calcein in FDR is initially combined with manganese ions and is quenched, but as amplification generates by-product pyrophosphate ions, these bind to and remove manganese from the calcein, resulting in fluorescence which is intensified further as calcein combines with magnesium ions [28,45]. On the other hand, if low molecular weight PEI is used, this forms an insoluble complex with high molecular weight DNAs, like LAMP products, which then captures the hybridized Rox-labelled probe into a pellet which fluoresces red under UV light [29]. All of our data confirmed that visual detection of assay results was compatible with the real-time turbidity measurement and agarose gel electrophoresis. Hence simple visual detection facilitates use of the assay in basic laboratories and in fish farms.

Compared with biochemical, microbial culture methods and PCR assay (24–48 hrs, 3 hrs respectively); the ERM-LAMP is convenient, rapid, and sensitive. The ERM-LAMP assay is 10-fold more sensitive than regular PCR as it detected a very low concentration of *Y. ruckeri *genomic DNA (1 pg), while the PCR can detect only till 10 pg *Y. ruckeri *genomic DNA. The assay successfully detected *Y. ruckeri *DNA in infected fish samples and hence appears suitable for use with clinical specimens.

## Conclusion

Loop mediated isothermal amplification assay as a new diagnostic tool for diagnosis of ERM disease in salmonids was developed and evaluated. The ERM-LAMP assay is rapid, as its result appeared after one hour, and sensitive than the conventional diagnostic method of ERM disease. The ERM-LAMP assay requires only a regular laboratory water bath and is hence suitable as a routine diagnostic tool in private clinics and field applications where equipment such as thermal cycling machines and electrophoresis apparatus are not available.

## Authors' contributions

MS carried out all the experimental work, data acquisition and drafted the manuscript. HS participated in the design of the study, analysis and interpretation of the data and helped to draft the manuscript. ME–M conceived and supervised the study, and revised the manuscript critically for important intellectual content. All authors read and approved the final manuscript.

## References

[B1] Raida MK, Buchmann K (2008). Bath vaccination of rainbow trout (*Oncorhynchus mykiss *Walbaum) against *Yersinia ruckeri*: Effects of temperature on protection and gene expression. Vaccine.

[B2] Austin B, Austin DA (1993). Bacterial Fish Pathogens: Disease of farmed and wild fish.

[B3] Furones MD, Rodgers CJ, Munn CJ (1993). *Yersinia ruckeri*, the causative agent of enteric redmouth disease (ERM) in fish. Ann Rev Fish Dis.

[B4] Rucker R (1966). Redmouth disease of Rainbow trout (Salmo gairdneri). Bull Off Int Epizoot.

[B5] Ewing WH, Ross AJ, Brenner DJ, Fanning GR (1978). *Yersinia ruckeri *sp. Nov., the redmouth (RN) bacterium. Int J Syst Bacteriol.

[B6] Bullock GL, Stuckkey HM, Shotts EB (1978). Enteric redmouth bacterium: comparison of isolates from different geographic areas. J Fish Dis.

[B7] Llewellyn LC (1980). A bacterium with similarities to the redmouth bacterium and *Serratia liquefaciens* (Grimes and Hennerty) causing mortalities in hatchery-reared salmonids in Australia. J Fish Dis.

[B8] Bragg RR (1991). Health status of salmonids in river systems in Natal (South Africa). III. Isolation and identification of bacteria. Onderstepoort J Vet Res.

[B9] Ross AJ, Rucker RR, Ewing WH (1966). Discription of a bacterium associated with redmouth disease of rainbow trout (Salmo gairdneri). Can J Microbiol.

[B10] Post G (1987). Text book of fish health.

[B11] Willumsen B (1989). Birds and wild fish as potential vectors of *Yersinia ruckeri*. J Fish Dis.

[B12] Coquet L, Cosette P, Junter GA, Beucher E, Saiter JM, Jouenne T (2002). Adhesion of *Yersinia Ruckeri *to fish farm materials: influence of cell and material surface properties. Colloids and surfaces B: Biointerfaces.

[B13] Stevenson RMW (1997). Immunization with bacterial antigens: Yersiniosis. Dev Biol Stand.

[B14] Austin DA, Robertson PAW, Austin B (2003). Recovery of a new biogroup of *Yersinia ruckeri *from diseased rainbow trout (*Oncorhynchus mykiss*, Wahlbaum). Syst Appl Microbiol.

[B15] Smith AM, Goldring OL, Dear G (1987). The production and methods of use of polyclonal antisera to the pathogenic organisms *Aeromonas salmonicida, Yersinia ruckeri*, and *Renibacterium salmoninarum*. J Fish Biol.

[B16] Tobback E, Decostere A, Hermans K, Haesebrouck F, Chiers K (2007). *Yersinia ruckeri *infections in salmonid fish. J Fish Dis.

[B17] Garcia JA, Dominguez L, Larson JL, Pederson K (1998). Ribotyping and plasmid profiling of *Yersinia ruckeri*. J Appl Microbiol.

[B18] Gibello A, Blanco MM, Moreno MA, Cutuli MT, Domenech A, Dominguez L, Fernandez-Garayzabal JF (1999). Development of a PCR assay for detection of *Yersinia ruckeri *in Tissues of inoculated and naturally infected trout. Appl Environ Microbiol.

[B19] Altinok I, Grizzle JM, Liu Z (2001). Detection of *Yersinia ruckeri *in rainbow trout blood by use of polymerase chain reaction. Dis Aquat Org.

[B20] Temprano A, Yugueros J, Hernanz C, Sanchez M, Berzal B, Luengo JM, Naharro G (2001). Rapid identification of *Yersinia ruckeri *by PCR amplification of yruI- yruR quorum sensing. J Fish Dis.

[B21] Compton J (1991). Nucleic acid sequence-based amplification. Nature.

[B22] Notomi T, Okayama H, Yonekawa T, Watanabe K, Amino N, Hase T (2000). Loop- mediated isothermal amplification of DNA. Nucleic Acids Res.

[B23] Zhang DY, Brandwein M, Hsuih T, Li HB (2001). Ramification amplification: A novel isothermal DNA amplification method. Mol Diagn.

[B24] Mori Y, Kitao M, Tomita N, Notomi T (2004). Real-Time turbidimetry of LAMP reaction for quantifying template DNA. J Biochem Biophys Methods.

[B25] Nagamine K, Hase T, Notomi T (2002). Accelerated reaction by Loop-mediated isothermal amplification using loop primers. Mol Cell Probes.

[B26] Mori Y, Nagamine K, Tomita N, Notomi T (2001). Detection of loop-mediated isothermal amplification reaction by turbidity derived from magnesium pyrophosphate formation. Biochem Biophys Res Commun.

[B27] Soliman H, El-Matbouli M (2005). An inexpensive and rapid diagnostic method of the koi herpesvirus (KHV) infection by loop-mediated isothermal amplification. Virol J.

[B28] Yoda T, Suzuki Y, Yamazaki K, Sakon N, Aoyama I, Tsukamoto T (2007). Evaluation and application of reverse transcription loop-mediated isothermal amplification for detection of noroviruses. J Med Virol.

[B29] Mori Y, Hirano T, Notomi T (2006). Sequence specific visual detection of LAMP reactions by addition of cationic polymers. BMC Biotechnol.

[B30] Kono T, Savan R, Sakai M, Itami T (2004). Detection of white spot syndrome virus in shrimp by loop-mediated isothermal amplification. J Virol Methods.

[B31] Mekata T, Kono T, Svan R, Sakai M, Kasornchandra J, Yoshida T, Itami T (2006). Detection of yellow head virus in shrimp by loop-mediated isothermal amplification. J Virol Methods.

[B32] Savan R, Igarashi A, Matsuoka S, Sakai M (2004). Sensitive and rapid detection of edwardsiellosis in fish by a loop-mediated isothermal amplification method. Appl Environ Microbiol.

[B33] Itano T, Kawakami H, Kono T, Sakai M (2005). Detection of fish nocardiosis by loop-mediated isothermal amplification. J Appl Microbiol.

[B34] El-Matbouli M, Soliman H (2005). Rapid diagnosis of *Tetracapsuloides bryosalmonae*, the causative agent of proliferative kidney disease (PKD) in salmonid fish by a novel DNA amplification method loop mediated isothermal amplification (LAMP). Parasitol Res.

[B35] El-Matbouli M, Soliman H (2005). Development of a rapid assay for diagnosis of *Myxobolus cerebralis *in fish and oligochaetes using loop-mediated isothermal amplification. J Fish Dis.

[B36] El-Matbouli M, Soliman H (2006). Development and evaluation of two molecular diagnostic methods for detection of *Thelohania contejeani *(Microsporidia), the causative agent of porcelain disease in crayfish. Dis Aquat Org.

[B37] Soliman H, El-Matbouli M (2005). Reverse transcription loop mediated isothermal amplification (RT-LAMP) for rapid detection of viral hemorrhagic septicaemia virus (VHS). Vet Microbiol.

[B38] Teng P, Chen C, Sung P, Lee F, Ou B, Lee P (2007). Specific detection of reverse transcription-loop-mediated isothermal amplification amplicons for Taura syndrome virus by colorimetric dot-blot hybridization. J Virol Methods.

[B39] Boehme CC, Nabeta P, Henostroza G, Raqib R, Rahim Z, Gerhardt M, Sanga E, Hoelscher M, Notomi T, Hase T, Mark D, Perkins MD (2007). Operational feasibility of using Loop-Mediated Isothermal Amplification for diagnosis of pulmonary tuberculosis in microscopy centres of developing countries. J Clin Microbiol.

[B40] Davies RL (1991). Virulence and serum-resistance in different clonal groups and serotypes of *Yersinia ruckeri*. Vet Microbiol.

[B41] Davies RL (1991). Outer membrane protein profiles of *Yersinia ruckeri*. Vet Microbiol.

[B42] Enosawa M, Kageyama S, Sawai K, Watanabe K, Notomi T, Onoe S, Mori Y, Yokomizo Y (2003). Use of loop-mediated isothermal amplification of the IS900 sequence for rapid detection of cultured *Mycobacterium avium *subsp. *Paratuberculosis*. J Clin Microbiol.

[B43] Thai HTC, Le MQ, Vuong CD, Parida M, Minekawa H, Tsugunori N, Hasebe F, Morita K (2004). Development and evaluation of a novel loop-mediated isothermal amplification method for rapid detection of sever acute respiratory syndrome Coronavirus. J Gen Virol.

[B44] Nagamine K, Watanabe K, Ohtsuka K, Hase T, Notomi T (2001). Loop-mediated isothermal amplification reaction using a nondenaturated template. Clin Chem.

[B45] Imai M, Ninomiya A, Minekawa H, Notomi T, Ischzaki T, Van Tu P, Tien NT, Tashiro M, Odagiri T (2007). Rapid diagnosis of H5N1 avian influenza virus infection by newly developed influenza H5 hemagglutinin gene-specific loop-mediated isothermal amplification method. J Virol Methods.

[B46] Karleson F, Steen H, Nesland J (1995). SYBR green I DNA staining increases the detection sensitivity of viruses by polymerase chain reaction. J Virol Methods.

[B47] Iwamoto T, Sonobe T, Hayashi K (2003). Loop-mediated isothermal amplification of *Mycobacterium tuberculosis *complex, *M. avium*, and *M. intracellulare *in sputum samples. J Clin Microbiol.

